# Towards a Sustainable One Health Approach to Crimean–Congo Hemorrhagic Fever Prevention: Focus Areas and Gaps in Knowledge

**DOI:** 10.3390/tropicalmed5030113

**Published:** 2020-07-07

**Authors:** Teresa E. Sorvillo, Sergio E. Rodriguez, Peter Hudson, Megan Carey, Luis L. Rodriguez, Christina F. Spiropoulou, Brian H. Bird, Jessica R. Spengler, Dennis A. Bente

**Affiliations:** 1 One Health Institute, School of Veterinary Medicine, University of California Davis, 1089 Veterinary Medicine Drive, Davis, CA 95616, USA; bhbird@ucdavis.edu; 2 Viral Special Pathogens Branch, Division of High-Consequence Pathogens and Pathology, Centers for Disease Control and Prevention, Atlanta, GA 30333, USA; pze7@cdc.gov (S.E.R.); ccs8@cdc.gov (C.F.S.); wsk7@cdc.gov (J.R.S.); 3 Department of Microbiology & Immunology, University of Texas Medical Branch, Galveston, TX 77555, USA; mncarey@utmb.edu (M.C.); dabente@utmb.edu (D.A.B.); 4 Galveston National Laboratory, University of Texas Medical Branch, Galveston, TX 77555, USA; 5 Huck Institutes of the Life Sciences, The Pennsylvania State University, University Park, PA 16802, USA; pjh18@psu.edu; 6 Foreign Animal Disease Research Unit, Plum Island Animal Disease Center, Agricultural Research Service, United States Department of Agriculture, Orient Point, NY 11957, USA; luis.rodriguez@usda.gov

**Keywords:** One Health, spillover, animal-human interface, Crimean–Congo hemorrhagic fever, tick-borne virus, outbreak response, surveillance, tick, livestock, risk reduction

## Abstract

Crimean–Congo hemorrhagic fever virus (CCHFV) infection is identified in the 2018 World Health Organization Research and Development Blueprint and the National Institute of Allergy and Infectious Diseases (NIH/NIAID) priority A list due to its high risk to public health and national security. Tick-borne CCHFV is widespread, found in Europe, Asia, Africa, the Middle East, and the Indian subcontinent. It circulates between ticks and several vertebrate hosts without causing overt disease, and thus can be present in areas without being noticed by the public. As a result, the potential for zoonotic spillover from ticks and animals to humans is high. In contrast to other emerging viruses, human-to-human transmission of CCHFV is typically limited; therefore, prevention of spillover events should be prioritized when considering countermeasures. Several factors in the transmission dynamics of CCHFV, including a complex transmission cycle that involves both ticks and vertebrate hosts, lend themselves to a One Health approach for the prevention and control of the disease that are often overlooked by current strategies. Here, we examine critical focus areas to help mitigate CCHFV spillover, including surveillance, risk assessment, and risk reduction strategies concentrated on humans, animals, and ticks; highlight gaps in knowledge; and discuss considerations for a more sustainable One Health approach to disease control.

## 1. Introduction

Tick-borne Crimean–Congo hemorrhagic fever virus (CCHFV; family *Nairoviridae*; genus *Orthonairovirus*) causes a severe, often fatal zoonotic hemorrhagic fever in humans, and is listed as a World Health Organization (WHO) and National Institute of Allergy and Infectious Diseases (NIH/NIAID) priority disease based on its capacity for person-to-person transmission, high mortality rate of the disease, and a lack of effective therapeutics or vaccines for human or animal use. Viral isolation and/or disease have been reported from more than 30 countries in Africa, Asia, Southeast Europe, and the Middle East. *Hyalomma* species ticks serve as both reservoir and vector of CCHFV; transmission to humans occurs through tick bite, crushing of engorged ticks, or, to a lesser degree, via contact with body fluids of domestic animals or patients with Crimean–Congo hemorrhagic fever (CCHF). Infection can result in a spectrum of disease; case fatality rates in outbreak settings range between 3% and 80%. Severe disease is characterized by a sudden onset of symptoms, such as high fever, headache, myalgia, and petechial rash, frequently followed by a hemorrhagic state and, occasionally, multiorgan failure [1].

Recently, new foci of CCHF have been identified in several parts of the world, including the Balkan countries, southwest Russia, the Middle East, India, and Spain. Potential reasons for the emergence or re-emergence of CCHF include anthropogenic factors, such as changes in agricultural activities, habitat fragmentation, and importation of infected animals and ticks [2,3]. The potential influence of climate change on the spread of the disease has also been suggested [4,5,6]. Outbreaks have historically been seasonal and sporadic with clusters of few cases. However, recent increases in the number of cases, especially in Turkey and southwest Asia, have demonstrated the imminent public health impact of this re-emerging disease [7,8]. The potential for zoonotic spillover to humans is high due to its wide geographic spread and persistent circulation in nature. In contrast to other emerging viruses, human-to-human transmission of CCHFV after spillover from the animal host or tick is limited, typically resulting in only small clusters of human disease. The importance of spillover for human disease supports prioritizing measures to mitigate these events.

**Figure 1:**
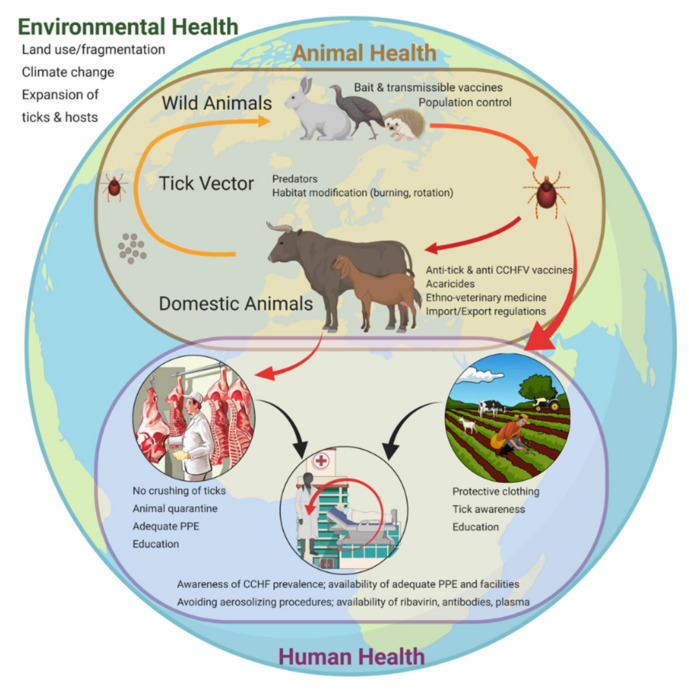
Crimean-Congo hemorrhagic fever virus (CCHFV) routes of transmission and associated intervention measures within the three realms of One Health. Environmental health (outer circle), including climate change and land use, influences several aspects of CCHFV transmission, including tick and animal host distribution and density; tick–animal host interactions affect the overall animal health status and livestock production levels (brown circle). The virus transmission pressure between ticks and animals increases progressively with the life cycle of the tick (reflected by the color gradient of the arrows from light orange to dark orange to red). Human health (purple circle) overlaps with the animal health realm through virus transmission routes, most commonly through tick bites or crushing of ticks, and secondarily through contact with viremic animal blood during the slaughtering process. Human-to-human transmission can also occur in a household or nosocomial setting when no proper personal protective equipment is worn.

The CCHFV life cycle involves silent transmission between multiple vertebrate hosts (wild and domestic) feeding immature or adult stages of the tick [9], with the absence of overt clinical disease in both hosts and ticks. This complex cycle, in which the hosts and vectors are by themselves highly influenced by environmental parameters, lends itself well to a One Health approach to disease prevention and control (Figure 1). The concept of One Health recognizes the interconnectedness of human, animal, and environmental health; it is an approach to disease prevention that endeavors to address human health in a broader context, making change and intervening in an interdisciplinary manner [10].

In 2019, a One Health-based framework entitled “A Tripartite Guide to Addressing Zoonotic Diseases in Countries” was published by The Food and Agriculture Organization of the United Nations (fao), the World Organization for Animal Health (oie), and WHO [11]. An important section of the guide focuses on taking a multisectoral One Health approach to disease control, including: (i) strategic planning and emergency preparedness; (ii) surveillance for zoonotic diseases and information sharing; (iii) coordinated investigation and response; (iv) joint risk assessment for zoonotic disease threats; (v) risk reduction, risk communication, and community engagement; and (vi) workforce development. Here, we examine critical topics for CCHF prevention, including surveillance, risk assessment, and risk reduction strategies for humans, animals, and ticks that could lead to a more sustainable One Health approach to disease control and outbreak response. In addition, by discussing several of the parameters and conditions that mitigate or exacerbate virus transmission, we highlight gaps in knowledge that, when addressed, would further support effective One Health control practices.

## 2. Surveillance

An effective surveillance system provides data on disease incidence and prevalence, establishes high-risk behaviors or practices at vector/host/human interfaces, and guides the development and implementation of preventive measures. Given the expansive geographic range of *Hyalomma* ticks, their multi-staged life cycle, and corresponding variability in host preferences, surveillance efforts must be coordinated across multiple sectors to inform on CCHFV in humans, animals, and ticks [2,12,13]. International surveillance for CCHF in humans comprises national surveillance programs in coordination with WHO regional offices, collaborating centers, and laboratories. Supplementing WHO-based surveillance networks are other organizations working in both the human and animal health sectors, including the FAO and OIE; non-governmental agencies, such as Médecins Sans Frontières and the Wildlife Conservation Society; and academic partners [14].

Since 2002, an increase in CCHF cases in Turkey resulted in greater awareness of the virus, leading to an influx of serology- and genome-based surveillance studies; over 35% of the CCHF publications in the last 15 years have focused on antibody and genome detection in humans, animals, and ticks (unpublished literature review by the authors). Despite substantial increases in data, many challenges in surveillance efforts remain, including the lack of a framework for extrapolating these data to formulate risk assessments in human populations. Below, we review international and country-specific approaches to surveillance in human, animal, and tick populations. We discuss current strategies and highlight remaining gaps in surveillance systems and methodologies. Detailed information on characteristics of specific diagnostic assays for CCHFV can be found in the WHO Roadmaps meeting report [15].

### 2.1. Human Surveillance

Five levels were recently proposed to reflect country-specific CCHFV surveillance systems [16]. Levels are based on the incidence of cases, potential for disease transmission to humans, and presence of surveillance systems. Level 1 countries are those in which human CCHF cases are reported annually and the virus is endemic; Level 2 countries have sporadic autochthonous human cases; Level 3 and 4 countries have no documented human cases but ecologic data, including the presence of *Hyalomma* ticks, suggests that cases may occur without detection; and Level 5 countries are ones for which no information is available. Human CCHFV surveillance strategies inherently vary between different country-specific levels. For example, Level 1 and 2 countries require a robust strategy, as human cases can be sporadic and thus public awareness may be lacking. In addition, these countries could have a relatively higher incidence of mild or asymptomatic human infections, which may occur more frequently than previously believed [17,18,19]. In contrast, Level 5 countries should focus surveillance specifically on the presence of the tick vector and not initially on detecting human seropositives [16].

While surveillance strategies will understandably vary based on the “level” of CCHFV in a country, wide gaps in the strength of human surveillance systems between countries with similar levels of endemicity remain [20]. Even across regions, surveillance approaches tend to lack standardization; their focus may be on acute outbreak-specific response, targeting at-risk populations, or examining larger groups of the population. Nasirian et al. (2019) recently performed a meta-analysis of 45 published human serosurveys, determining that animal contact, husbandry, and farming, as well as tick bites, hunting, and slaughtering were the most evident risk factors for CCHF seropositivity [20]. This report serves as an example of the power of collective analysis of national and international reports; however, the utility of this approach would be greatly enhanced with improved standardization of data collection and reporting.

Although human serosurveillance can be used to generate these types of high-level risk assessments, data should be interpreted with caution. Important gaps in CCHF knowledge are the discrepancy between seroprevalence and symptomatic disease incidence and an understanding of CCHFV strain-specific virulence. More specifically, we find that seropositivity in human populations cannot be assumed to definitively correlate with a risk for human CCHF disease. For example, within Greece, CCHFV was first isolated from a *Rhipicephalus bursa* tick in 1975 (strain AP92/P7) [21]. Subsequent human surveillance data have indicated widely varying rates of seropositivity in different regions within Greece, some as high as 27.5%, yet only one recorded human case of CCHF has been documented within the country since 1975 [22]. It has been hypothesized that the Greek AP92 strain is an outlier, confined only to Greece and avirulent or less virulent than other strains circulating worldwide, though laboratory studies have never been conducted to confirm these hypotheses. However, in recent years, AP92-like strains have been discovered outside of Greece, and interestingly, these genetically similar viruses have been implicated in causing severe human disease [23,24].

Adding to disparate surveillance networks and methodologies are unknowns regarding the accuracy and cross-reactivity of serologic testing. No standard serologic assay has been approved for human clinical use, and the assays used for research purposes have shown variation in performance based on genetic heterogeneity of the virus [25,26,27]. Thus, human surveillance data alone cannot be used to understand risk in a given geographic area. Rather, a more comprehensive One Health-based approach to CCHF surveillance should be used to provide a more refined understanding of virus spillover risk.

### 2.2. Animal Surveillance

An extensive number of serosurveys have been performed in domestic and wildlife species informing on both the geographic range and relative levels of CCHFV circulation [28,29]. Animals are fundamental to virus maintenance by serving as hosts for the ticks; they develop a subclinical infection with a relatively low-level viremia typically lasting a week and up to 14 days [30]. The duration of viral replication in other tissues over time has not been characterized in livestock and wild-life species. However, in mouse models of disease, RNA levels in survivors indicate that virus may be detected in certain tissues longer than in blood [31,32], indicating a need for both tissue tropism and viral persistence studies in livestock and other animals.

Serological data are the mainstay in animal surveillance for CCHFV [28]. PCR is of limited value; infectious virus is found only briefly in animals as they have a short period of viremia, and while virus may persist longer in tissues, this period is not thought to be extensive. This brief persistence is reflected in the small number of reported virus isolates obtained directly from animals and the large sample size required to obtain virus isolates [29,33,34,35]. As such, PCR has limited sensitivity for identifying animals involved in outbreaks or maintenance cycles, even if an exposure event was relatively recent. In contrast, while serology cannot indicate active infection, it can identify past exposure and is not affected by the sensitivity limitations inherent in PCR analyses for CCHFV in animals.

Serological data can be very useful for revealing the geographic range of CCHFV but have limitations. Critical details (e.g., species, breed, age, proportion of herd sampled, sampling dates, etc.) are often lacking, necessitating more standardized methodology and reporting. As with human data, serology in animals must also be interpreted with caution. First, without repeated sampling, these data do not indicate the timing of exposure, emphasizing the importance of longitudinal sampling in a region. While human data support long-lived antibody and CD8+ T-cell-mediated responses [36,37,38], the antibody responses to virus in livestock (and many other species) have not yet been characterized over time. In experimentally infected mice, anti-CCHFV antibodies generally developed around one week post inoculation (p.i.); IgM appeared, beginning at 8 days p.i. and declined thereafter, whereas IgG levels increased and were sustained at least 9 weeks p.i. Only marginally neutralizing antibody responses were detected by day 28 p.i., and did not substantially increase by 9.5 weeks p.i. [31]. Adding to the complexity, wildlife and livestock are most likely exposed to infected ticks multiple times throughout a tick season and their lifespan. It is unknown how this influences isotype-specific antibody levels over time. Longitudinal sampling in individual animals may be prohibitive from a surveillance standpoint, but experimental data on antibody persistence can inform the frequency of sampling decisions based on the level of animal turnover in the area.

The movement of animals can also complicate serological data interpretation, as the location in which the sample was collected may differ from the location of exposure. Furthermore, maternal transfer of anti-CCHFV antibodies has been demonstrated in sheep [39], suggesting that even if offspring have not moved, the geographic history of the dam may also have to be examined. Finally, cross-reactivity to other nairoviruses should also be considered, as sequence homology has been described between members of different serogroups, including the Nairobi sheep disease serogroup, known to circulate and cause disease in domestic animals [40,41]. Cross-reactivity between nairoviruses has also been reported in protein expression assays [42]. To date, the degree of cross-reactivity between nairoviruses has not been systematically evaluated; evidence suggests that it may occur, but the frequency and level of cross-reactivity are unknown.

### 2.3. Tick Surveillance

Ticks are the vector and reservoir of CCHFV, maintaining the virus in nature through transstadial and transovarial transmission [29,43,44,45,46]. Ticks within the genus *Hyalomma* (family Ixodidae) are considered to be the primary vectors and exhibit a two-host lifecycle, in which ticks in larval and nymphal stages feed on small mammalian hosts and later switch to a different larger mammalian host as adults to complete their life cycle [1]. Evidence has demonstrated that the geographic distribution of *Hyalomma* ticks specifically overlaps with human CCHF cases, which is not the case for other tick species, suggesting that the presence of *Hyalomma* ticks may be necessary to support natural circulation of the virus [29,47,48,49]. While the role of other tick species in CCHFV maintenance and transmission is not well defined, the virus has been detected in several other tick genera (*Amblyomma*, *Rhipicephalus*, *Dermacentor*, and *Ixodes*); however, data on the vector competence of these species is largely missing [47]. Assessing vector competence is crucial to understanding the potential for CCHFV establishment in new geographic regions, particularly those where non-*Hyalomma* species might supplement the transmission cycle or play a role in cryptic transmission [50].

*Hyalomma* ticks are broadly distributed across Asia, Europe, and Africa, making the potential for CCHFV circulation and spillover a widespread global concern. Additionally, changing climatic conditions and the frequent importation of *Hyalomma* ticks into novel regions via migratory birds or animal trade (e.g., livestock) is of particular concern for the expansion of endemic areas [51,52,53,54]. The detection of viral antigen or genomic fragments in tick vectors has historically been an important precedent and/or predictor of human cases. For example, in 2010, prior to any reports of human cases in Spain, CCHFV RNA was detected in that country in *Hyalomma lusitanicum* and *Hyalomma marginatum* ticks at a rate of 2.78% (44/1579), which was similar to the CCHFV tick infection rate of other endemic countries in Europe, including Kosovo, Bulgaria, and Albania [3,55,56]. These data were important in confirming the established spread of CCHFV into western Europe 7 years before the first human case was documented in Spain in 2017 [55,56]. Additionally, viral antigen was detected in ticks in Iran over 20 years before autochthonous human cases were described [57]. Studies that focus on tick collection from cattle can be a particularly sensitive indicator of the presence of viral circulation in a given geographic area because cattle can be infested by a greater density of *Hyalomma* spp. ticks than small ruminants [58].

Although baseline detection of virus in ticks is important, many other factors that we do not fully understand determine the long-term maintenance and persistence of the virus in ticks over time, including rates of transstadial and transovarial transmission, co-feeding behaviors [59], and relative densities of both ticks and animal hosts. In order to better understand the frequency and contribution of transstadial/transovarial transmission in the maintenance of CCHFV in tick populations, surveillance efforts should focus on collecting questing ticks, though this can be significantly more difficult than collecting ticks from animal hosts. Recently, a growing number of publications have reported the detection of CCHFV in non-*Hyalomma* tick species collected from animal hosts, suggesting that these species may play a role in transmission to humans [47]. It must be emphasized that the detection of virus in non-questing ticks, particularly those collected from animal hosts, cannot be correlated with vector competence or risk for transmission to humans. Vector competence ultimately needs to be evaluated and confirmed in a laboratory setting.

## 3. Risk Assessment

**Figure 2:**
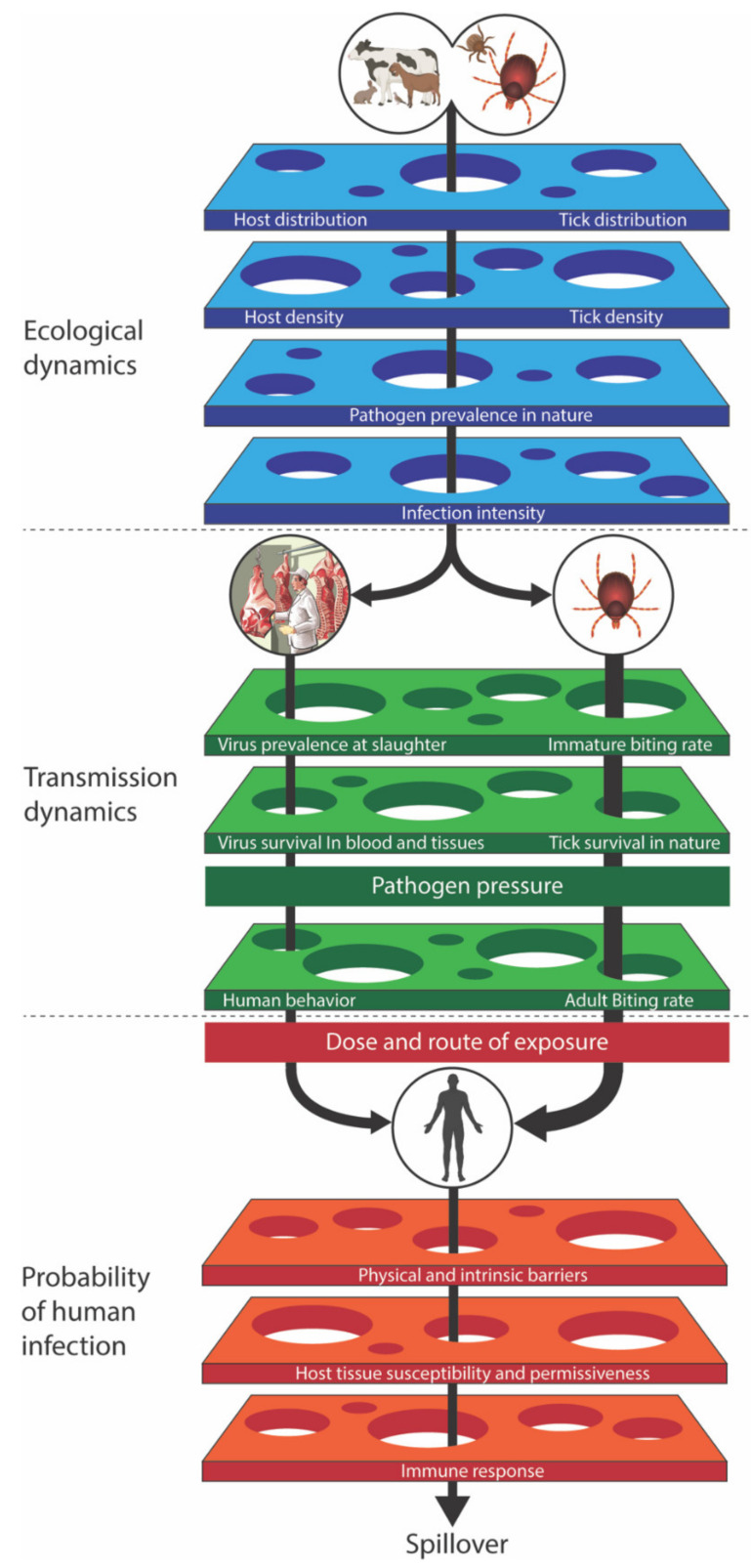
Spillover percolation model of different barriers to CCHFV spillover and opportunities to intervene (based on Plowright et al., 2017) [61]. The risk of CCHFV spillover is determined by the ecological dynamics of infection in the tick and animal host (blue barriers), human epidemiology/behavior (green barriers), and within-host biological factors (orange barriers) that influence exposure outcome in humans. CCHFV must overcome all of the barriers before spillover can occur; thus, each barrier offers intervention opportunities to prevent human disease. The distribution and intensity of infection (virus levels) in tick vectors and animal hosts, virus release from ticks and animal hosts, and CCHFV and tick survival determine the pathogen pressure (the amount of virus that is available to humans at a given point in time and space). Pathogen pressure then interacts with human behavior and the tick vector to determine the likelihood, dose, and route of human exposure. The thickness of the arrows represents the likelihood of spillover to humans (tick > livestock-associated exposure). The probability of human infection is determined by the within-host factors. Once spillover has occurred, human-to-human transmission in a household or nosocomial setting can occur as well (not depicted). In this case, barrier parameters, such as virus dose, route, and human behavior, still apply.

Although many of the individual determinants leading to spillover, such as virus prevalence in ticks or animals, have been studied extensively, each is typically addressed in isolation within its own discipline. As a result, prevention is often focused on interventions targeting specific aspects of transmission without accounting for other interconnected parameters. This can lead to unproductive intervention efforts (as described in Section 6.3), such as with the release of helmeted guineafowl [60]. A comprehensive understanding of virus maintenance in nature and spillover into human populations, and how these processes are hierarchically, functionally, and quantitatively linked remains a fundamental deficit in CCHF research. Plowright et al. (2017) proposed a mechanistic structure of determinants of disease spillover and their interactions [61]. We propose that this comprehensive framework be adopted to guide research efforts in support of a One Health-based approach for CCHF (Figure 2). In addition to considerations regarding epidemiology, ecology, virology, and vector biology (covered in other sections of this manuscript), mathematical modeling will play an important role in adopting the framework to develop comprehensive risk assessments. Modeling within the context of the framework will serve to highlight key gaps in knowledge, which will facilitate prioritization of epidemiology and laboratory-based experiments and of mitigation policies.

### 3.1. Modeling Tick-Borne Viruses

Modeling of tick-borne viruses has historically been lacking, primarily due to the complexity of the vector-host-virus system. Mathematical models can be used to integrate virus, vector, and host biology in order to identify key features that increase the risk of infection. These models are little more than a formal description of our biological understanding, but they reveal the non-linearities (a lack of a direct relationship between an independent variable and a dependent variable) associated with transmission and identify major shifts in transmission dynamics that can result in unexpected outbreaks. Working from the tick life cycle and adding key features of CCHFV biology, we can start to identify critical data needed to estimate outbreak risk; we also determine whether an understanding of the system in one location helps to explain the findings in another, and what aspects of the system shape transmission. Ultimately, we seek to obtain insights into the dynamics of the virus in relation to the vector and hosts, which will lead us to identify the type of surveillance that can provide critical information for developing targeted intervention strategies to reduce infection risk in humans and livestock.

Tick models are fundamentally different from mosquito models because ticks bite once per developmental stage, live over multiple seasons, and frequently feed on multiple host species, some of which are capable of transmitting the virus on to other ticks (amplifying hosts) while others are not. There are multiple non-linearities in the system for which the output does not change in direct proportion to a change in the input: The rate of transmission rises as a consequence of host–tick combinations and the resulting heterogeneity of how CCHFV is amplified in this system, leading to transmission and outbreaks driven by certain vector-host combinations. In this respect, models of tick-borne infections are probably best used to determine if our understanding of the biology explains the changes in seroprevalence from year to year in different host species and variation between locations. Additionally, models can be useful in differentiating competing hypotheses and validating models by making predictions that can be tested with critical experiments (Figure 2). Indeed, such experiments frequently provide important data to improve parameter estimation (i.e., finding the unknown parameters of the model that give the best fit to a set of experimental data), and model precision and accuracy.

In some types of analysis, multiple spillover determinants are aggregated, obscuring the interactions between them. Although aggregation might be appropriate at times, sometimes discrete mechanisms can only be identified using simpler models. Non-linear interaction between the spillover determinants and barriers can create bottlenecks that provide opportunities for One Health interventions.

### 3.2. What Key Data Are Needed?

In endemic areas, seroprevalence in hosts is most likely in an equilibrium and is roughly constant from year to year since livestock hosts are repeatedly exposed to the virus through ticks in various life stages persistently infected with CCHFV. Young CCHFV-naïve animals join the population as old animals die. Therefore, examining seroprevalence across the age curve over years can be some of the most helpful data. This curve quite simply plots the proportion of hosts within each age category that have been exposed to infection and tracks the changes in this proportion with host age. With directly transmitted freely mixed populations, these data can be used to obtain an estimate of the basic reproduction number, *R*_0_, which would be 1 + (*L*/*A*), where *L* is the life expectancy of the host and *A* is the average age of infection [62]. *R*_0_ is useful when there is no previous exposure in the population and when comparing different studies, and is defined as the number of secondary cases in a population of susceptible hosts that arises following the introduction of an infectious individual. *R*_0_ for many vector-borne systems is not simple to estimate but provides a rough approximation that can be very useful for comparing populations and then validating with a more precise metric.

Cooper (2007) applied a basic tick-protozoan model of *Theileria parva* developed by Medley et al. (1993) to CCHF disease dynamics between ticks and their domestic animal hosts [63,64], and clearly showed how seroprevalence data can be used to estimate the rate at which animals become infected. In this situation, the most useful study would be long-term longitudinal sampling of livestock of a known age so that the time of seroconversion can be cleanly estimated. In the absence of longitudinal studies, detailed sampling across the age structure at one time point can provide interesting data for comparison between sites, assuming the seroprevalence has reached equilibrium.

Ak et al. (2018) developed a computational framework based on Gaussian processes to spatiotemporally predict human cases in Turkey. The predictive value of the framework was tested against CCHF cases in the years 2004 and 2015 [65]. This Gaussian process framework obtained better results than two frequently used standard machine learning algorithms (i.e., random forests and boosted regression trees) under temporal, spatial, and spatiotemporal prediction scenarios. This shows that such frameworks have the potential to make an important contribution to public health policy makers.

### 3.3. Important Features of CCHF Modeling

Several features of the CCHF-tick-host system could generate non-linear responses in transmission and result in disease outbreaks. Here, we mention a few, although there is a pressing need to understand how some of these processes could produce rapid changes in exposure risk. In several instances in which data regarding *Hyalomma* spp. were unavailable, we consider what is known about other ixodid ticks and tick-borne diseases. We acknowledge that translating information from other tick or tick-borne diseases may not be an accurate approach, since the tick and pathogen life cycle are significantly different from those involved in maintaining CCHFV, but it may be a first step in helping frame ideas for the control of CCHF.

#### 3.3.1. Multiple Host Species and the Dilution Effect

Modeled by Norman et al. (1999) [66], the dilution effect occurs when the transmission cycle, maintained by one competent vertebrate host species is reduced by the presence of a second host species that is less competent. The dilution effect has been demonstrated for Lyme disease but is still a controversial hypothesis for other tick-borne diseases. Adding a second host species to the community of ticks effectively increases the number of vertebrate hosts available to feeding ticks so the tick population expands. If this second vertebrate host is less competent for the virus, the virus is lost from the system: The presence of the second host dilutes the virus. This is because ticks feed a limited number of times during their life cycle, and feeding on the less competent host effectively means reduced virus transmission. This results in fewer infectious vertebrates within the population and a lower *R*_0_ value for the virus in that host community, even though the *R*_0_ for the ticks is effectively increased. Field studies on louping ill virus have shown that removing the amplifying host (in this instance, a non-viremic host) results in declining viral abundance in line with the theoretical models [67]. Excellent studies have also been undertaken on the dilution effect of Lyme disease [68].

#### 3.3.2. Non-Viremic Transmission through Co-Feeding

In the case of most vector-borne viruses, transmission occurs when the arthropod vector bites a viremic host and becomes infected. However, some hosts permit direct passage of CCHFV between co-feeding ticks without the requirement of a viremic response from the host. During feeding, ticks produce pheromones that attract other ticks to the same feeding pool, facilitating tick-to-tick transmission, a process accelerated by the presence of tick saliva [69]. The likelihood of co-feeding increases with the intensity of tick infestation, and hosts infested with high numbers of tick larvae and nymphs are more likely to have co-feeding groups. The relative importance of co-feeding transmission in wildlife hosts preferred by *Hyalomma*, like *Leporidae*, is not yet known [70]. Thus, more field studies looking at the densities of *Hyalomma* species ticks on wildlife hosts need to be conducted (Figure 2). The co-feeding transmission phenomenon has been well characterized for tick-borne encephalitis virus (family Flaviviridae; genus *Flavivirus*) and *Ixodes* spp. ticks, yet only one publication addresses this question for CCHFV. A small proportion of ticks (0.1–1.9%) were shown in a laboratory study to acquire CCHFV when co-feeding on non-viremic guinea pigs [70]. However, guinea pigs are not the natural host for *Hyalomma* ticks, nor do they play a role in the natural transmission of the virus. Thus, co-feeding transmission between ticks must be much better characterized in studies with natural hosts in a controlled laboratory setting (Figure 2).

#### 3.3.3. Transovarial Transmission

Passing the virus from the ovaries of female ticks to the egg and eventually the larva is the mechanism for maintaining viral persistence from one generation to the next. Indeed, infecting a high proportion of larval ticks can also result in increased *R*_0_. This has been recorded for CCHFV in *Hyalomma truncatum*, although at a fairly low level [71]. The role of transovarial transmission in different *Hyalomma* species is unclear and needs to be investigated in a controlled laboratory environment (Figure 2).

#### 3.3.4. Sexual Transmission of the Virus in Ticks

Gonzales et al. (1992) showed sexual transmission of CCHFV between copulating *Hyalomma truncatum* ticks, although the sample size was insufficient to reliably estimate the rate of transmission. Since virus-induced mortality is unlikely in ticks, the prevalence of infection is expected to rise from one tick life stage to the next, so sexual transmission is a route by which host infection and transmission of CCHFV to the eggs can increase [71].

#### 3.3.5. Role of Other Tick Species: Vector Competence and Vectorial Capacity

Current knowledge indicates that *Hyalomma* ticks are the primary vector of CCHFV transmission, but in many locations, other tick species are present and may contribute to transmission. Vector competence (the ability to become infected with and transmit a virus) of ticks needs to be tested further to improve our understanding of which species and life stages are important in CCHFV ecology [47]. Besides vector competence, we must also understand vectorial capacity, which is the amount of transmission that occurs. For example, a highly competent vector that rarely feeds on infected hosts or has an extrinsic incubation period (time from virus ingestion to transmission) longer than the time between feedings is unlikely to cause much onward transmission. In contrast, a poorly competent tick vector can initiate an outbreak if it prefers to feed on competent mammalian hosts and has an extrinsic incubation period shorter than the time between feedings. Furthermore, the extrinsic incubation period depends on molecular interactions within the tick, such as the microbiome of the tick gut, cross-immunity with other viruses, and the tick immune response [72], as well as external factors like co-feeding responses and interactions with host susceptibility. Understanding vector competence and vectorial capacity is vital in predicting CCHFV expansion to new geographic areas and must be investigated further (Figure 2).

## 4. Risk Reduction: Human-Targeted Approaches

### 4.1. Physical and Chemical

Occupations at high risk for CCHF include veterinarians, abattoir workers, and farmers. For veterinarians and abattoir workers, the use of standard infection control practices when handling potentially infectious blood or ticks aids in risk reduction [73]. Farmers accounted for almost 90% of CCHF cases during a recent outbreak in Turkey [73], emphasizing environmental exposure to ticks as a major source of virus transmission. Basic methods to reduce tick bites, such as wearing appropriate clothing (e.g., long sleeves, pants, etc.), chemical approaches (e.g., repellent), and visual inspection of skin and clothing will contribute to decreased transmission risk. It is noteworthy that few tick repellents have been definitively evaluated for use against *Hyalomma* ticks, and that *Hyalomma* adults have been shown to have a greater affinity for human hosts than other species [29,74,75,76,77], potentially reducing the relative efficacy of these approaches for CCHF disease control compared to other tick-borne pathogens.

When attached ticks are found, proper tick removal techniques should be used; improperly removing attached ticks can crush them, spilling infected blood and facilitating virus transmission [78]. Thus, effective risk communication and community education promoting proper tick inspection and removal techniques are vital. The most effective methods for safely removing the whole tick including the mouthparts, while also minimizing the risk of tick-borne disease transmission, are mechanical and can be performed with tools readily available in most regions [78,79]. Specifically, mechanical removal with tweezers (82.5% success rate) was found to be superior to both lassoing (47.5% success rate) and card detachment (7.5% success rate) [79].

Other considerations for tick transmission of CCHFV are: (i) the amount of virus transmitted; (ii) time to transmission post attachment; and (iii) potential enhancement of infection by tick saliva. Unlike other pathogens (e.g., *Borrelia* spp. [80]), the kinetics of CCHFV transmission by *Hyalomma* ticks are not known. Ebel and Kramer (2004) demonstrated that Powassan virus (family Flaviviridae; genus *Flavivirus*) could infect mice within 15 min of tick attachment [81]; this has shaped the dogma that all tick-borne viruses are transmitted immediately after tick attachment. However, a study in mice with Dugbe virus (genus *Orthonairovirus*) demonstrated the absence of viral antigen in the salivary glands of newly attached *Amblyomma* ticks [82]. This indicates that virus transmission in ticks may not always occur as rapidly as suggested in earlier Powassan virus studies. Studies are needed to characterize CCHFV-specific transmission kinetics. However, regardless of the exact timing of virus transmission, prompt tick removal must be emphasized to minimize the risk of transmission.

### 4.2. Behavior

Human–animal interactions differ across cultures depending on the species of animals present, views towards domestic animals and ticks, and cultural perceptions as a whole. The nature of these interactions may result in activities that increase the risk of CCHFV exposure. Social and cultural practices, particularly those that include the movement of potentially infected animals, have been epidemiologically linked to CCHF outbreaks [83,84,85]. For example, the practice of livestock (cattle, sheep, goat, or camel) sacrifice central to festivals like the Hajj and Eid-al-Adha results in the contact of large numbers of people with potentially infectious animal blood and body fluids. During Eid-al-Adha, nearly 8 million animals are sacrificed each year in Pakistan alone [84]; 2 million small animals and 750,000 cattle are slaughtered in Turkey, accounting for 25% of all annual slaughtering in that country [84].

The risk of exposure to pathogens like CCHFV during slaughtering is known, and practices have been implemented to reduce exposure during these festivals. In general, individuals performing the sacrificial rite during the Hajj or Eid-al-Adha should adhere to careful techniques, including slaughter at designated sites and sanitary collection and disposal of blood and tissues [9]. In Iran, viral hemorrhagic fever surveillance protocols are in place and report various slaughtering statistics, including the location of slaughtering (i.e., by industrialized, smaller slaughterhouses or by individuals). During Eid, cities coordinate with the Iran Veterinary Organization and create special temporary slaughtering centers. The Saudi Ministry of Health actively discourages amateur slaughtering by encouraging the use of “ritual coupons”, allowing the Hajjees to fulfill their sacrificial obligation by proxy without performing the act themselves. Instead, the Saudi Project for Utilization of Hajj Meat performs the ritual with the supervision of government and veterinary workers and then distributes the meat to the poor according to the Muslim tradition. This process could be extended to further mitigate virus transmission to the public. Furthermore, if Hajj authorities banned importation of sacrificial animals from CCHF-endemic countries and instead mandated ritual coupons for travelers, there would be added protection against virus transmission to those making the Hajj. With this plan in place, Muslims from CCHF-endemic countries could still faithfully fulfill their Hajj obligation while minimizing the risk of spreading the virus to non-endemic regions and reducing the chances of an outbreak in Saudi Arabia. Acaricides and quarantine (14 days) are another option for reducing CCHFV transmission during these festivals [83,86]. If acaricides are unavailable or if rates of resistance are high, a mandatory 4-week quarantine for all animals imported for slaughter from CCHF-endemic countries can help mitigate exposure risk. The first 2 weeks would allow any attached ticks to drop off the animal, and the second 2 weeks would allow any active viremia to subside [83].

### 4.3. Vaccination

A WHO Research and Development Blueprint for Action to Prevent Epidemics working group established a draft roadmap analysis for the CCHF field and outlined a timeline for both benchmarks and deployment goals for a human vaccine [15]. They supported refining the top contending experimental vaccines with a target product profile and promoted clinical safety and early immunogenicity trial testing of selected vaccines by 2019, with phase II trials encouraged for 2023. The draft roadmap analysis prioritizes further animal model development to establish correlates of protection, which can satisfy regulatory authorities for use in pre-clinical evaluations and regulatory approval for human CCHFV vaccines [15].

To date, several CCHFV vaccine candidates have been developed [87,88] with a variety of antigenic variations (strain and/or gene combinations). Vaccine evaluation has been performed using different dosing schemes that involve diverse adjuvants, inoculation routes, challenge strains, and prime/boosts strategies [88,89]. In animal models, vaccination has been shown to generate humoral immunity and can generate up to 100% protective efficacy depending on the platform and approach to vaccination. For the last decade, disease models of CCHFV have been limited to immunocompromised mouse strains [89,90]. The most effective antigen component(s) and correlates of protection for CCHFV vaccines are not yet clear. When used in experimental vaccines, the nucleoprotein generates a more T-cell based response, while other viral antigens, such as whole or portions of the glycoprotein precursor, can generate potent immunoglobulin responses [88,89,90]. Passive transfer of sera and T-cells generated to either the nucleoprotein or glycoprotein molecules also did not protect but delayed time to death [91,92,93]. Other studies have implicated that antibodies targeting certain portions of the glycoprotein neutralized CCHFV but also failed to provide protection in vivo [94,95]. Conversely, antibodies targeting non-structural elements of the glycoprotein precursor can produce non-neutralizing protection in animal models [94,95,96].

To our knowledge, only one study has examined human humoral protective responses to CCHFV vaccination, demonstrating antibody induction with low neutralizing capacity and a T-cell-induced immunity after multiple booster doses [97]. Taken together, the data indicate that experimental vaccines eliciting both robust T-cell-driven responses to nucleoproteins and glycoproteins, in combination with targeted serological responses to non-structural elements of the glycoprotein precursor, are the ideal candidates to move forward in clinical trials. However, without a much clearer understanding of correlates of protection in humans, relying on correlates of protection from immunocompromised mice can confound the knowledge needed to develop a successful human vaccine. A recent non-human primate model has demonstrated a spectrum of disease, from asymptomatic to severe, mirroring that observed in humans [98]. However, the severe stages of disease from this model have not been wholly reproducible, as other groups have demonstrated [99] and clinical measures of protection must be established for the model to aid in developing countermeasures, such as vaccines. Additional challenges to vaccine development include strain variance (designing a vaccine that is efficacious across the diverse array of geographic CCHFV clades) [1] and assessing safety profiles for experimental vaccines. Furthermore, the sporadic incidence of human cases, often in rural areas lacking reliable infrastructure for cold chain transport, will impede the return on investment for vaccine manufacturers.

## 5. Risk Reduction: Animal-Targeted Approaches

### 5.1. Wild Animals

Controlling human diseases shared with wildlife is complex but can be achieved by different means, including: (i) preventive actions; (ii) arthropod vector control; (iii) host population control through random or selective culling, habitat management, or reproductive control; and (iv) vaccination [100]. Preventive actions (translocation control, barriers, husbandry, etc.) are fundamental approaches for disease control in domestic animals and wildlife but are challenging for CCHFV due to the wide array of host species involved in virus ecology. Notably, the composition of vertebrate hosts on which *Hyalomma* spp. feed is different than for other tick genera. Specifically, immatures feed preferentially on species of the orders Lagomorpha and Rodentia and the class Aves, while adults concentrate mainly on members of the family Bovidae [9]. Thus, efforts to control virus transmission in wildlife will predominantly disrupt transmission by immatures but do not necessarily disrupt virus maintenance by adult *Hyalomma* ticks.

Vector control methods (e.g. acaricides and vaccines) provide viable options for CCHF prevention as discussed later (see Section 6). Population control (random culling, selective culling, habitat modification) may also be considered in endemic areas, as the population density of recognized hosts has been directly associated with the incidence of human disease. For example, *Leporidae* spp. are central in the sylvatic cycle of *Hyalomma* ticks and act as amplifying hosts for immature ticks and CCHFV [9]. Importantly, their population densities have been directly correlated with CCHF incidence. However, population control often consists of an intervention in natural ecosystems and, as such, is often controversial [101]. More socially acceptable alternatives can be expensive and prohibitive based on access to animals; availability of convenient, sensitive, and specific tests; prevalence of infection; and spatial distribution of the target population [100].

Despite presenting its own challenges, wildlife vaccination is another viable option and is proposed by Monath as a Framework III vaccine [102]. A vaccination program targeting vast populations of highly reproductive species over large geographic regions is ambitious. However, tick-borne diseases, including CCHF, are almost always found in geographically circumscribed foci. Therefore, geospatially focused control efforts guided by preceding serosurveillance are a viable option. Some of the barriers to implementing wild animal vaccination include: (i) involvement of multiple species in natural transmission cycles; (ii) safety concerns for non-target species; (iii) high reproductive rates and population turnover; (iv) fastidious feeding behaviors and difficulty in designing effective baits; (v) delivery difficulties due to very high or, conversely, very low population densities of the target species; (vi) environmental concerns and concerns about release of genetically modified organisms; (vii) difficulty in designing an effective formulation for oral immunization; (viii) instability of a vaccine or vector under prevailing environmental conditions; and (ix) requirement for low unit cost and government funding for vaccine purchase and delivery [102]. However, vaccines preventing wildlife diseases have been successfully developed to reduce public health impacts in human populations (e.g., vaccination against rabies of raccoon, coyote, fox, skunk, and bat; against bovine tuberculosis in Eurasian badger), reduce economic effects on the livestock industry (e.g., vaccination against brucellosis in bison and elk; against classical swine fever in wild boar), and address concerns for wildlife conservation (e.g., vaccination against sylvatic plague in black-footed ferret and prairie dog; against white-nose syndrome in hibernating bats) [103]. Oral vaccination is the most common method of wildlife vaccination because it does not require capture and has been proven successful for protecting raccoons, foxes, and coyotes against rabies [104], thereby reducing the risk of rabies exposure in other wild and domestic animals and humans.

The most sustainable approach to eradicating a zoonotic disease from an environment is a reservoir-targeted vaccine (RTV). RTVs have the potential to be effective at breaking the transmission cycle of many tick-borne pathogens including, but not limited to, *Borrelia burgdorferi*, *Borrelia miyamotoi*, *Borrelia mayonii*, *Babesia microti*, and *Anaplasma phagocytophilum* [105]. Multiple studies have shown that free-ranging white-footed mice, the reservoir hosts of Lyme disease, can be effectively vaccinated via RTV bait boxes and that the vaccination both decreases *B. burgdorferi* uptake by larval ticks and decreases transmission from infected ticks to their natural mouse reservoir [105,106,107]. A similar approach can be envisioned for CCHF, though *Hyalomma* ticks are the long-term reservoir. Decades of effort have shown that targeting non-feeding ticks in the environment either with chemical or physical means does not efficiently reduce tick population density. Therefore, a reservoir-targeted approach focusing on the amplifying hosts of the immature stages of *Hyalomma* ticks, *Leporidae*, would be most successful. A two-pronged approach to RTV that could both interrupt CCHFV transmission and reduce tick populations would likely be most effective.

Non-transmissible vaccines are limited by the quantity and scale of product delivery, whereas transmissible vaccines that can spread from one individual to the next allow much broader coverage with equivalent dosing. A transmissible vaccine must have two crucial properties: A live vectored vaccine must be able to establish and propagate in a given host population, and it must remain attenuated during transmission between vertebrates. A transmissible vaccine with these properties would have numerous benefits that could overcome the challenges of strategies relying on direct vaccination. Ideally, once introduced into a given population, transmissible vaccines would increase herd immunity more efficiently then direct vaccination [108]. However, while transmissible vaccines offer many advantages, serious drawbacks must be addressed in vaccine design or vaccination schemes. These include the potential for reversion to virulence or to inducing clinical signs of disease, short-lived immunity for some pathogens, instability during storage, and contraindications in some subpopulations (e.g., in pregnant animals) [109].

### 5.2. Domestic Animals

As noted above, livestock, mainly of the family Bovidae, are preferred hosts for adult *Hyalomma* ticks. The livestock–tick and livestock–human interface provide additional opportunities to reduce virus transmission. Operational decisions and livestock preventative health programs are affected by the awareness of risks associated with emergent and endemic diseases of both animals and humans [82]. Although CCHFV infection control may not have perceived animal health benefits, several approaches can reduce virus exposure in animals (and, in turn, humans) and have positive outcomes beyond CCHFV risk reduction. Decreasing the tick burden on animals by physically removing ticks (see Section 4.1) and using acaricides (see Section 6.2) can improve the overall health status of an animal or herd and increase production levels [110,111]. These are significant concerns to herd health, so efforts are made to target breeding programs for tick resistance [112].

Due to limitations in the advancements of a human vaccine against CCHF, the WHO draft roadmap provides alternative vaccination strategies that may be more cost-effective and sustainable at controlling disease, including the development of animal vaccines. Veterinary vaccine development is far less costly; overall, the price of veterinary vaccines, from bench to market, is only ~10% of that of human vaccines. Additional benefits compared to human vaccines include a far less stringent regulatory framework and the ability to directly evaluate candidates in target species. Animal vaccines, however, have their own unique limitations. For example, livestock may require a much larger vaccine dose compared to humans, making certain vaccine platforms (e.g., DNA, RNA, or virus-like particles) extremely cost-prohibitive, and others (e.g., replication-competent platforms or subunit vaccines) are more appropriate for use.

Monath’s Framework II vaccines are defined as those that protect humans either indirectly by interrupting transmission of viruses amplified by domesticated animals or directly by preventing virus spread from infected animals to humans, for example, during the slaughter process for CCHFV [102]. The advancement and success of Framework II vaccines for bacterial (*Brucella abortis*, *E. coli* O157) and viral (rabies, influenza, West Nile, Rift Valley fever, and Hendra viruses) pathogens [102] could be capitalized on to develop a multi-valent platform that addresses one or more of these pathogens in conjunction with CCHFV, thus increasing demand.

An important consideration in vaccine design for use in animals is the ability to differentiate previously infected animals from vaccinated animals (DIVA). To accomplish this, the vaccine would need some type of identifiable marker (either a marker fused to the antigen or a deleted portion of the antigen itself) to limit confounding in seroprevalence studies by vaccinated animals. One example of the DIVA principle is in foot-and-mouth disease (FMD) vaccine preparations [113]. These preparations lack non-structural FMD viral proteins. Serological assays detecting nonstructural protein reactivity can therefore be used to indicate previous infection with wild-type virus as opposed to vaccination. CCHFV also encodes non-structural viral glycoproteins, suggesting that a similar approach could be used. However, it is important to note that despite the ideal goal of implementing DIVA practices for transboundary animal diseases, such as CCHFV, designing and evaluating a DIVA vaccine, with the goal of maintaining a high degree of sensitivity, can ultimately increase the total time, cost, and efforts of development. These facets may curtail the benefits offered by a DIVA animal vaccine and are important factors when considering prevention options.

Similar to wildlife (see Section 5.1), RTVs would be a desirable vaccine approach in domestic animals. A two-pronged design conferring anti-tick and anti-CCHFV immunity would be most effective due to simultaneous control of both the virus and the virus reservoir. It is also conceivable that CCHFV vaccines could be added on to conventional common animal vaccines, such as those to prevent bovine herpes virus or lumpy skin disease [114]. In the end, a vaccine will need to be developed that is safe, effective, easy to use, and cost effective. A conceivable approach to a cost-effective strategy could be delivering the immunogen through the feed as shown by using a transgenic tobacco plant expressing CCHFV glycoprotein [115]. As we move to develop safe and effective candidates, we must keep in mind the importance of strategies that aim to increase both vaccine access and demand, all of which are largely dependent on the involvement of donors and international organizations to stimulate and facilitate sustainable adoption [116].

## 6. Risk Reduction: Tick-Targeted Approaches

### 6.1. Physical

Manual habitat modification for tick control can affect both immature and adult ticks. For example, the removal of vegetation that shelters immature ticks can reduce their population levels [117]. In addition, physical modification of tick habitats can be used to control adult ticks at the livestock interface. Tick densities decline rapidly in areas where grazing lands are rotated with crops, removing large animal hosts from the accessible range [118] and thus disrupting CCHFV transmission and maintenance by adult ticks. Studies investigating overall tick burden have found that a single spelling/rotation period annually is thought to be promising if combined with acaricide treatments in an integrated pest management approach [119]. However, rotational grazing strategies (frequent movement of cattle between different pens) in the presence [117] and absence [119] of acaricide treatment have also been shown to be effective. Pasture management, including rotational grazing of cattle in Australia and in Zambia, as a tick control strategy is believed to be responsible for an overall decrease in tick burdens on livestock animals [120,121]. Investigating these approaches in areas with a high density of *Hyalomma* ticks would be helpful in determining whether or not they should be promoted in the prevention and control of CCHF.

### 6.2. Chemical

The use of synthetic acaricides on livestock animals has long been a favored methodology for ectoparasite and tick control throughout the world [122]. Organophosphates are the major chemical acaricides used for ectoparasite control and include compounds, such as pyrethroids, macrocyclic lactones, and amidines, among others [123]. Acaricides are a comparatively inexpensive method for tick control and can be easily administered in multiple ways [124], including dipping, footbaths, or manual sprayers. They vary in efficacy, cost effectiveness, sustainability, and risk to the workers [125]. The ongoing and indiscriminate use of acaricides, however, has led to the development of resistant tick populations, and in recent years, several countries have reported almost complete resistance to most acaricides, presenting a worldwide challenge for ongoing successful tick control [126,127,128,129,130]. Although concerning, resistance in *Hyalomma* ticks has not yet been tested; thus, it is unclear to what extent resistance may contribute to decreased control of *Hyalomma* populations. Additionally, acaricidal compounds can have off-target effects, including toxicity to other organisms, and long-term environmental persistence [131]. For these reasons, the discovery and development of new synthetic acaricidal compounds has not been an actively pursued area of research in recent years [132].

More recently, the focus has been on developing alternative environmentally safe tick control strategies that can circumvent the development of resistance. Historically, many rural farming communities have used plants or plant extracts to control ticks on livestock. The efficacy of some of these ethnobiological methods has been explored in the literature and a recent review. Using in vitro assays, Adenubi et al. (2016, 2018) found that over 200 plant species from several countries around the world demonstrated tick-repellent or acaricidal activity, and some of these also showed marked in vitro acaricidal activity comparable to the efficacy of many synthetic acaricides currently used [133,134]. However, there are several limitations to these studies: (i) a lack of standardized testing methods makes comparisons between studies difficult to interpret and extrapolate for use in controlling ticks on animals; (ii) in vitro data predominate in the literature, and may not adequately recapitulate the efficacy of plants or their extracts in field trials; and (iii) compounds may not persist in the environment due to degradation caused by photo-oxidation, temperature, pH levels, and microbial action [135]. Moreover, differences in cultivating and collecting plant materials (including variations in soil, climate, and other factors) for plant or extract production may affect downstream results [136]. Despite the limitations of current data, based on their historic use by rural livestock farmers, plant-based compounds may be a good future source of effective acaricidal preparations either as an extract or as a source of new synthetic acaricidal compounds [132,134].

### 6.3. Biological

Due to the limitations of synthetic (and naturally derived) acaricides for tick control, alternative biological methods have been explored, including the use of natural tick predators and pathogenic bacteria, viruses, and fungi. Ants, beetles, and spiders are the major arthropod predators of ticks [137]. Overall, the use of arthropods to reduce tick populations is impractical due to the difficulty in large-scale reproduction and use across broad geographic areas. Birds have been anecdotally documented as capable of preying on ticks, and early evidence from a 1992 study suggested that birds could control ticks carrying Lyme disease in the United States [138]. However, studies have since shown that birds are more likely to play a role in disseminating ticks and their pathogens than in controlling them [51,53,54,139,140]. The systematic breeding and release of birds to control *Hyalomma* tick populations was in fact implemented in Turkey in 2011 and clearly demonstrated the inherent flaws of this strategy. Thousands of helmeted guineafowls (*Numida meleagris*) were introduced to decrease the circulation of CCHFV and its tick vector, but the birds consumed negligible numbers of ticks and instead served as intermediate hosts facilitating the expansion of the *Hyalomma* tick population [60].

Pathogenic bacteria, viruses, and fungi have also been evaluated for their ability to control arthropod populations [141,142,143]. Fungi showed the most potential and have been effectively used to control medically and agriculturally important arthropods, including German cockroaches and cotton stainer bugs [144,145,146]. Fungi have been developed industrially with well-established application and dosing methods for commercial use against arthropods [144,145,146]. *Metarhizium anisopliae* is the most well-studied fungal pathogen of arthropods and is considered to particularly promising due to posing minimal risk to non-target organisms. *M. anisopliae* has been shown to effectively control nymphal *Ixodes* tick [147], cattle tick [148,149,150], and *Hyalomma* tick populations by inducing high mortality in immature stages and decreasing reproductive fitness in females [151].

Currently, the OIE and the International Research Consortium for Animal Health (STAR-IDAZ) has significant interest in developing anti-tick vaccines to combat a range of tick-borne pathogens. Unlike other tick control measures, the immunological control of ticks is exempt from environmental problems and may provide a broader prevention measure by reducing targeted tick populations in general. Anti-tick vaccines are developed against one of two different types of antigenic targets: “Exposed” antigens that in nature are presented to the animal’s immune system (e.g., proteins or peptides secreted in the tick’s saliva during attachment and feeding on the host), or “concealed” antigens not normally visible to the host immune mechanisms but associated with a vital function for the tick (e.g., structural components of the tick gut).

To date, efforts to develop anti-tick vaccines have focused on controlling the cattle tick *Rhipicephalus microplus* due to its economic importance. Currently, no anti-tick vaccines based on exposed antigens are commercially available, although some research has been done in this area. Two commercial cattle tick vaccines are available (TickGARD and Gavac), both based on the concealed antigen Bm86, the midgut membrane-bound protein of *R. microplus* [152]. Immunization induces antibodies in the vertebrate host; once ingested, the antibodies interfere with the biological function of Bm86, leading to a reduction in the number, weight, and reproductive capacity of engorged female ticks. Cross-protective efficacy of TickGARD and Gavac has been demonstrated in cattle against *Hyalomma dromedarii* and *Hyalomma anatolicum* ticks (but not *Rhipicephalus appendiculatus* or *Amblyomma variegatum*). The tick antigen subolesin (4D8) was found to be conserved between representatives of six different genera of ticks, including *Hyalomma* [153], offering the prospect for broader cross-species tick vaccines.

Another option for CCHFV control would be vaccinating animals to target the virus in the tick and at the point of entry into the host. Bm86-based vaccines have been shown to induce host antibodies that are ingested when ticks feed on the vaccinated animals and enter the tick gut, thereby causing damage. Thus, it is tempting to speculate that the tick could take up antibodies against CCHFV from appropriately vaccinated animals, potentially resulting in virus neutralization that would prevent virus dissemination to the tick salivary glands or eggs. Such a vaccine may have the potential to reduce infection in difficult to vaccinate hosts (e.g., wild animals), as feeding ticks may be prevented from transmitting the virus.

Experience to date suggests the need to design vaccines tailored to specific local tick populations or to seek universal tick antigens to use as immunogens. Anti-tick vaccines capable of reducing virus transmission will need to be effective against *Hyalomma marginatum*, other *Hyalomma* species, and other competent tick vectors. Existing vaccines and vaccine candidates may also offer some promise. As CCHFV transmission has been demonstrated during co-feeding of infected and naïve ticks on naïve animals [70], decreasing the overall amount of ticks that can acquire virus could play a role in decreasing the spillover events for this disease. However, further research is required to identify potential protective *Hyalomma* antigens together with proof-of-concept studies to demonstrate their efficacy in experimental animals and in the field.

## 7. Conclusions

The global incidence of CCHF has been rising as changing climatic conditions have led to increased vector survival and expanded disease ranges into previously unaffected geographic regions [4,5]. Additionally, land and habitat fragmentation has been a key factor in exposing animal hosts and humans to tick populations [2]. Although acaricide resistance had not been definitively evaluated in *Hyalomma* ticks, the growing resistance of many tick species to acaricides in endemic countries is also hypothesized to have contributed to higher densities of CCHFV vectors. These factors, compounded by intensified contact between people and livestock, all greatly increase the risk of virus spillover into human populations. The current distribution of the virus across much of Asia, Europe, and Africa is likely to continue to spread over time and poses a significant health risk wherever it may be found. Therapeutic options for treating disease are limited and, in the absence of a human vaccine, alternative approaches to preventing spillover to human populations are essential.

CCHFV spillover events are the summation of environmental, ecological, anthropogenic, and viral genetic factors that must align to result in human disease. The large number of contributors makes predicting future spillover events challenging; however, they also provide opportunities for intervention. We believe that a tiered approach outlining these factors will help to develop a more effective understanding of spillover risk (Figure 2). Factors that include the distribution, density, and infection prevalence in ticks and their animal hosts are the baseline data forming the foundation for targeted risk assessments. Beyond these basic data are human behaviors that predispose certain populations to tick or livestock-associated exposure. Even after virus has breached the physical and intrinsic barriers of the body, disease does not always occur. The virus must be able to replicate effectively (a function of both host and virus genetics) and sufficiently antagonize and evade the immune response. There are numerous gaps in our knowledge of CCHFV in regard to these parameters, necessitating investigations to develop a clear picture of disease risk within defined geographic populations (Table 1).

Given the complexities of CCHFV ecology and endemic circulation as outlined here, we believe that a multisectoral One Health approach that integrates human and animal health, vector biology, and environmental health will be essential in addressing and mitigating the threat of CCHFV in the future. First, building a strong surveillance network of veterinarians, physicians, and epidemiologists can provide important knowledge regarding baseline prevalence of virus in humans, animals, or tick vectors in a given geographic region. Second, as discussed, these data become more powerful when analyzed by modelers and ecologists who can build it into quantitative data-driven risk assessments. Lastly, the data from these risk assessments can be utilized by national One Health networks to target specific tick, animal, or human populations for preemptive interventions that make the most sense for the given population at the given time.

Although not addressed here, the importance of cultivating a national One Health workforce in individual countries cannot be underestimated. In order to adequately integrate data across sectors and implement many of the measures discussed in this paper, a workforce must be developed to carry out and sustain these activities on a national, regional, and individual level. The Tripartite Guide to Addressing Zoonotic Diseases in Countries includes step-by-step recommendations for building the multisectorial One Health network and infrastructure necessary to collect and integrate data, expertise, and personnel from all sectors (environmental, veterinary, medical, etc.) [11]. This process involves activities, such as identifying and convening stakeholders, reviewing current in-country surveillance information, uncovering workforce gaps, developing educational and training programs to address these gaps, and developing a national cohesive strategy for addressing disease.

Ultimately, the way in which data on CCHFV surveillance, risk assessment, and reduction strategies are shared, interpreted, and acted upon must be regionally specific and defined by an interpretation of available data across sectors and the availability of financial resources. While financial resources may be a significant limitation in some countries or sectors, in the long term, many One Health interventions tend to be cost effective and result in improved public health outcomes, decreasing the economic impact that may occur when certain populations are affected by a disease, (e.g., agricultural workers in the case of CCHF). Additionally, coordination across the human, animal, and environmental health sectors can help to reduce costs by avoiding duplication of activities. The economic benefits of improved public health can be used to justify further investment in disease mitigation and aid in supporting a sustained One Health approach to CCHF prevention.

## Figures and Tables

**Table 1:** Gaps in knowledge of CCHFV and factors contributing to disease. Key considerations and questions regarding parameters of viral maintenance and transmission, including both host and tick factors, that contribute to spillover events (as depicted in Figure 2). These questions aim to highlight areas warranting additional investigation. Reports addressing a subset of these questions currently exist; however, the questions are listed to indicate that more data are required to adequately inform prevention and control efforts.

Parameter	Key Considerations and Questions to Address Gaps in Knowledge of CCHFV
Tick distribution	Which tick species are competent vectors and what is their vectorial capacity? What are the molecular determinants of vectorial competency? Where are these species found and how is their distribution changing? How do migratory birds, climate change, animal movement, and land fragmentation influence tick range expansion or appearance in new areas? What is the risk of CCHFV introduction into new geographic areas? Are there additional cryptic transmission cycles of which we are not yet aware?
Host distribution	Which hosts are more prominent in virus maintenance? Which are competent at transmitting CCHFV to ticks, and how well do they transmit virus? Which hosts are amplifiers of key tick species? What host density is needed to support virus maintenance?
Tick density	What factors affect immature and adult tick population density? Can we develop methods to quantitatively assess *Hyalomma* in the field? How can we predict increases in tick density? What tick density is needed to support virus maintenance? What is the relationship between tick density and spillover/outbreak events?
Host density	What is the relationship between host density and spillover/outbreak events? Does host diversity and dilution effect play a role for virus maintenance?
Virus prevalence in nature	How has the virus adapted to support the maintenance cycle in ticks and non-human mammals? What is the relative prevalence of virus in tick populations? What is the relative incidence of infection in host species/populations? What are the best measures of pathogen prevalence? What are the sensitivity and specificity of serological assays?
Infection intensity *	Is viremia in host animals necessary to maintain virus in nature or is co-feeding transmission sufficient to maintain the virus in nature? What is the duration and level of viremia in different host species? What are transstadial and transovarial transmission rates? What is viral load and tropism within the tick?
Virus prevalence at slaughter	What is the prevalence of virus infection in slaughtered animals? What is the virus load in tissues of infected animals? How do levels change over time? How stable is the virus in blood and animal tissues?
Biting rate (immatures)	How relevant is co-feeding transmission? How efficient is co-feeding transmission and how often does it occur?
Virus survival in blood and tissues of livestock	How long does infectious virus persist in blood and tissues? What are the relative levels of infectious virus in tissues? (I.e., handling of which tissues pose the highest risk?)
Tick survival in nature	How does climate change influence tick life stage survival?
Human behavior	What are high risk activities/groups? What is the level of disease awareness in high risk groups? How widely do individuals in high risk groups accept (or adhere to) preventative measures?
Biting rate (adults)	What is the affinity of the tick species for biting humans?
Dose and route of exposure	What is the infectious dose? What is the rate of subclinical infection? Does exposure route alter clinical course or outcome? Are human-to-human transmitted virus strains more virulent than tick-transmitted strains?
Physical and intrinsic barriers (e.g., skin)	What are the contributions of mucosal and dermal immunity in viral pathogenesis? What is the stability of virus on skin and mucous membranes? How does tick saliva influence pathogenesis and virulence? How quickly is virus transmitted from ticks?
Host tissue susceptibility and permissiveness	What are underlying risk factors in humans? Genetic factors (e.g., HLAs, polymorphisms in immune response)? How do pre-existing conditions affect susceptibility to infection/disease? What is virus tropism in the host and what factors influence it? What is the relative virulence between virus strains? What are the viral determinants of virulence? How does CCHFV suppress the immune response?
Immune response	What is the contribution of the innate immune response to disease? Does mitigating the immune response aid or exacerbate disease progression? What role do innate and adaptive responses play in protection? I.e., what are the correlates of protection?

* Infection intensity refers to the average quantity of virus present in an infected host or tick.
